# Supplementary material for: Targeting the MEK/ERK Pathway to Suppress P-Glycoprotein and Reverse Carfilzomib Resistance in Multiple Myeloma
Source: Int J Mol Sci. 2025 Nov 26;26(23):11448. doi: 10.3390/ijms262311448 (PMC12692345; doi:10.3390/ijms262311448)

Figure S1. Relative (**a**) *CROT* and (**b**) *IL6R* genes expression in AMO-1 and AMO-1/CFZ cells after exposure to IC_{50} of cobimetinib (COB) or IC_{50} of ulixertinib (ULIX) for 72 and 96 h. Gene expression was quantified by RT-qPCR and normalized to housekeeping gene *RPLPO*. The values are presented as the ratio between cells treated with inhibitors and control cells from five experiments. Statistical analysis was conducted using ANOVA multiple comparisons test with unpaired Student's t-test with Welch's correction. * - p<0.05,** - p<0.01, *** - p<0.001, **** - p<0.0001

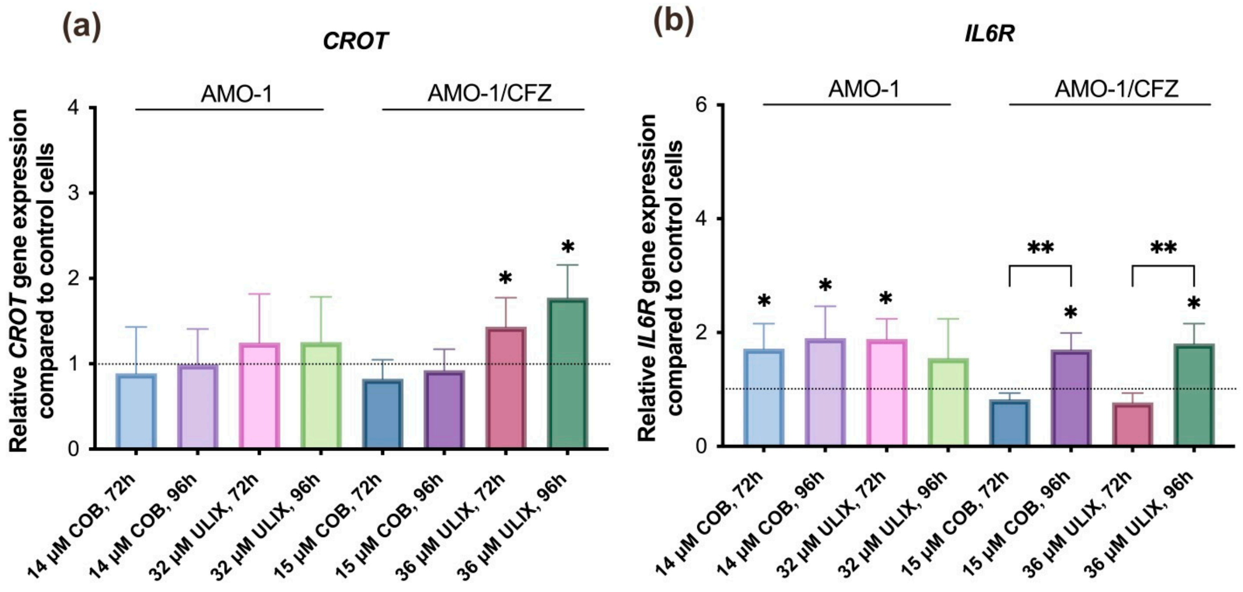

Figure S2. Molecular docking of several known and potential inhibitors to P-glycoprotein (PDB code: 6QEX). (a, b) Well-known third-generation P-glycoprotein inhibitors elacridar (green) and tariquidar (orange) in 3D structure, radial position in the molecule; (c) The first-generation P-glycoprotein inhibitor verapamil (yellow) in 3D structure, radial position in the molecule; (d) Potential inhibitor ulixertinib (pink) in 3D structure, radial position in the molecule; (e) Potential inhibitor cobimetinib (blue) in 3D structure, radial position in the molecule.

(a)

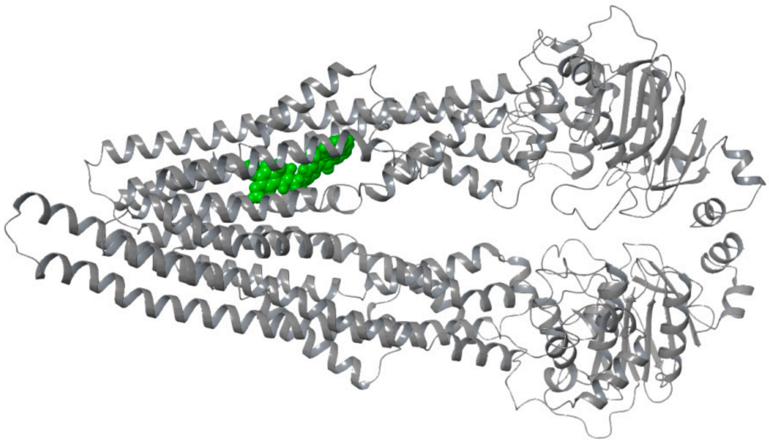

(b)

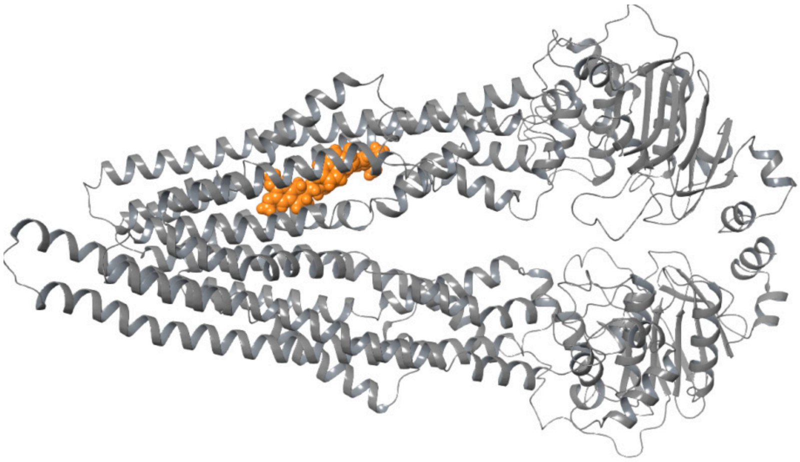

(c)

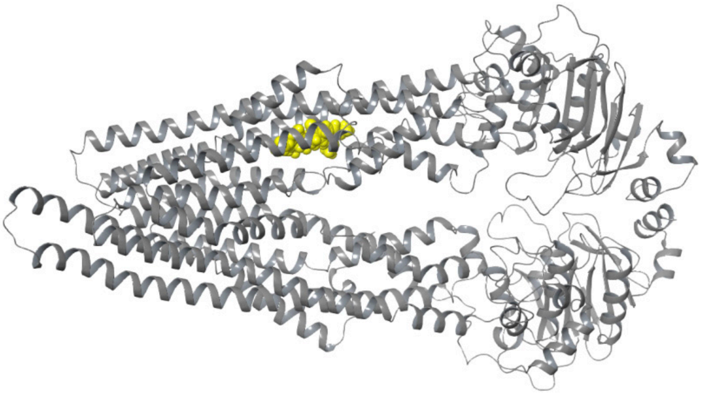

(d)

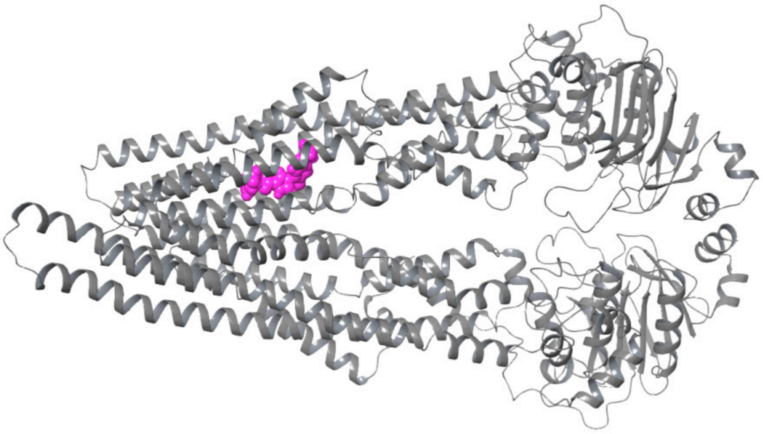

(e)

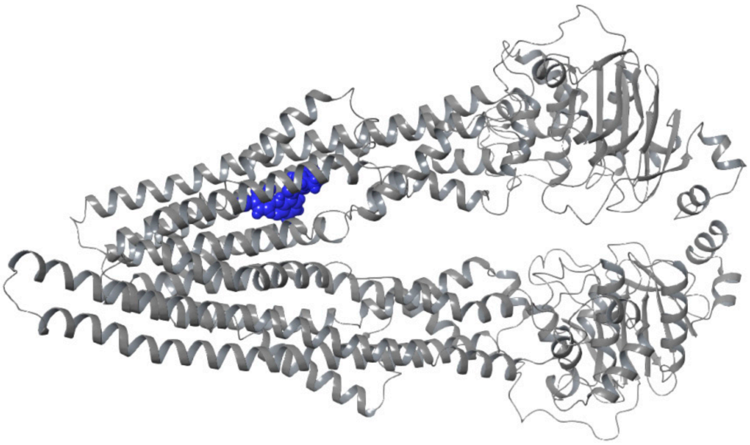

Figure S3. Molecular docking of proteasome inhibitors to P-glycoprotein (PDB code: 6QEX). (a) Bortezomib (light pink), radial position in the molecule; (b) Ixasomib (red), radial position in the molecule.

(a)

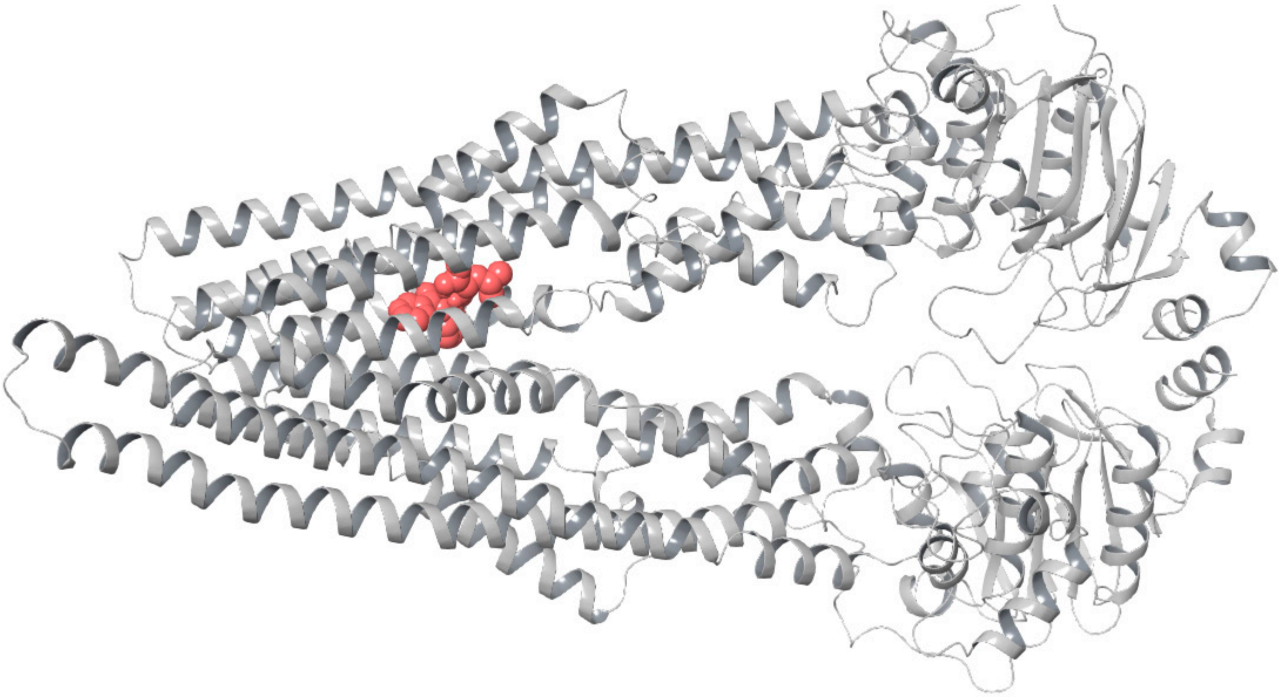

(b)

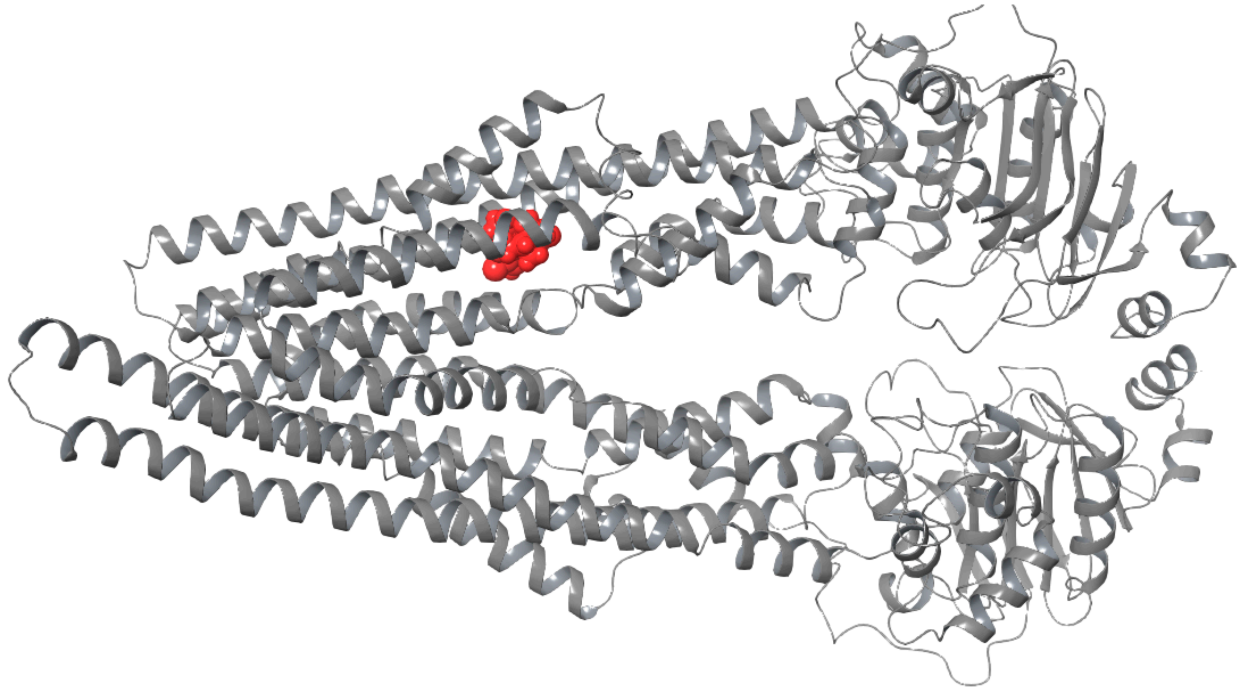

Figure S4. Molecular docking of several known substrates to P-glycoprotein (PDB code: 6QEX). (a) Carfilzomib (yellow) in 3D structure, central position in the molecule; (b) Well-known substrate of P-gp paclitaxel (blue) in 3D structure, central position in the molecule; (c) Well-known substrate of P-gp doxorubicine (pink) in 3D structure, central position in the molecule; (d) Well-known substrate of P-gp rhodamine 123 (light pink) in 3D structure, central position in the molecule.

(a)

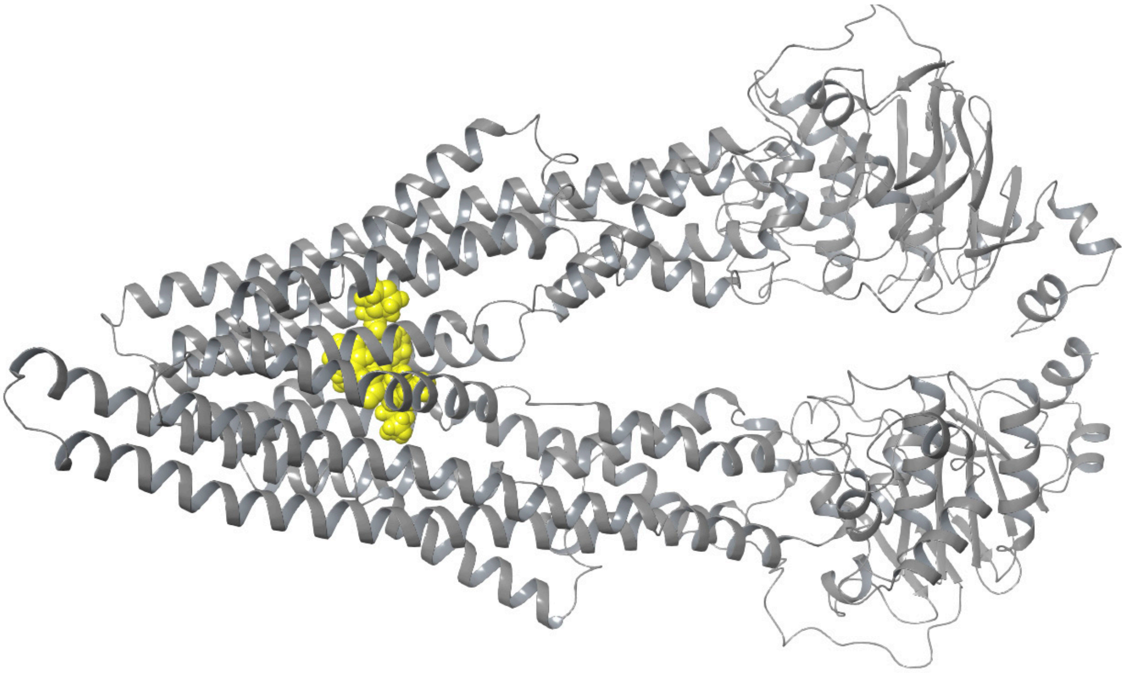

(b)

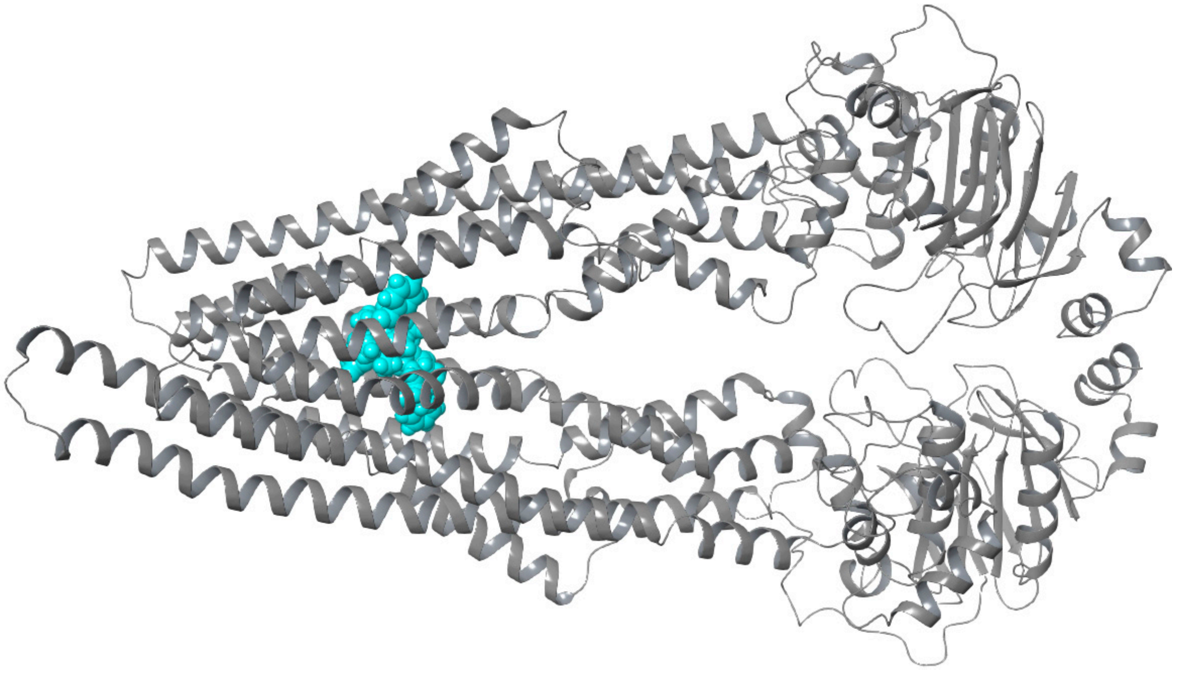

(c)

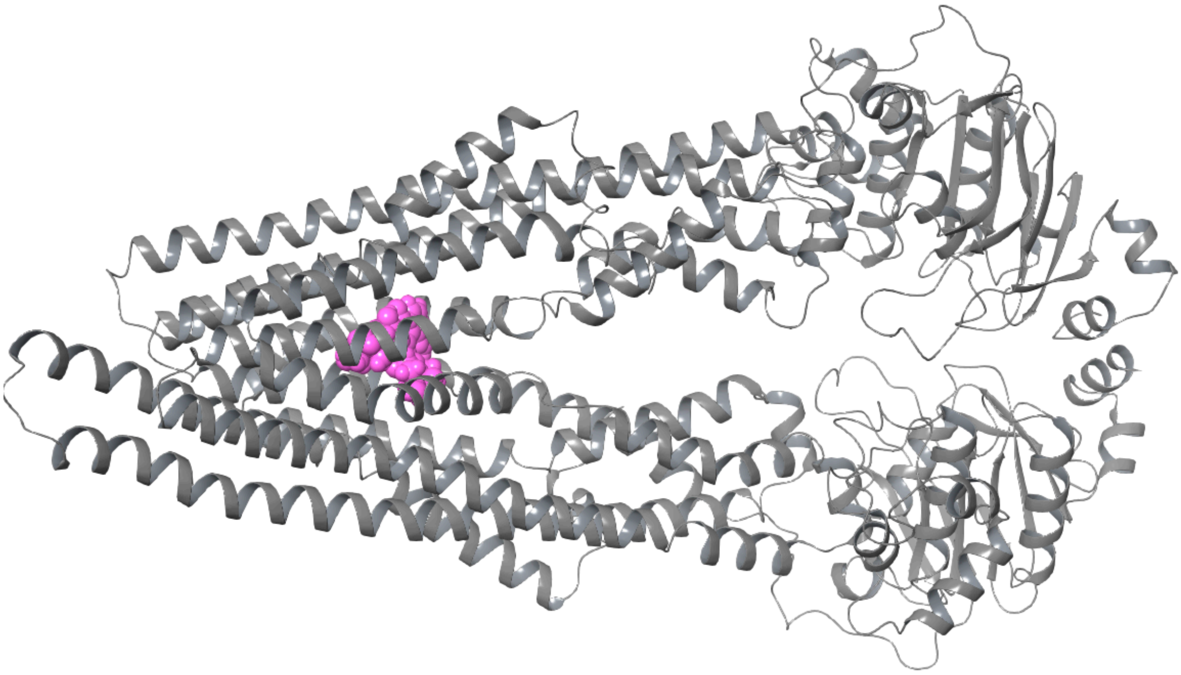

(d)

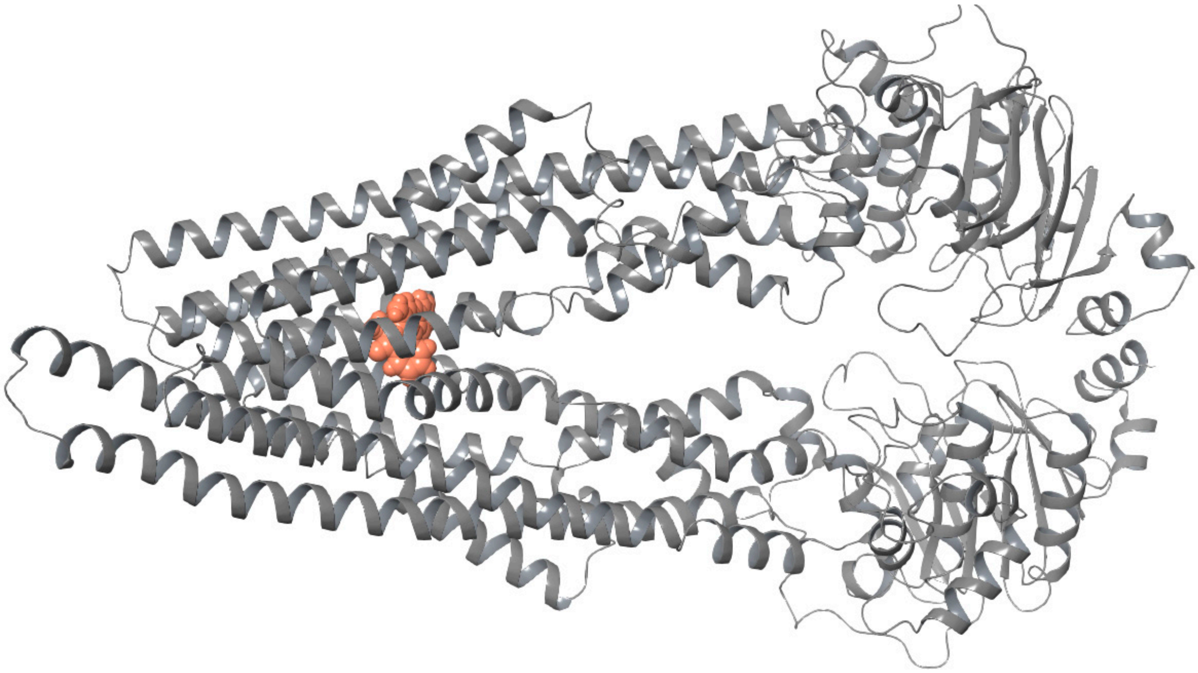

Figure S5. Two-step molecular docking of the known and potential P-gp inhibitors and carfilzomib to P-glycoprotein (PDB code: 6QEX). (a) Carfilzomib (black) and elacridar (green) in 3D structure; (b) Carfilzomib (black) and ulixertinib (purple) in 3D structure; (c) Carfilzomib (black) and cobimetinib (blue) in 3D structure.

(a)

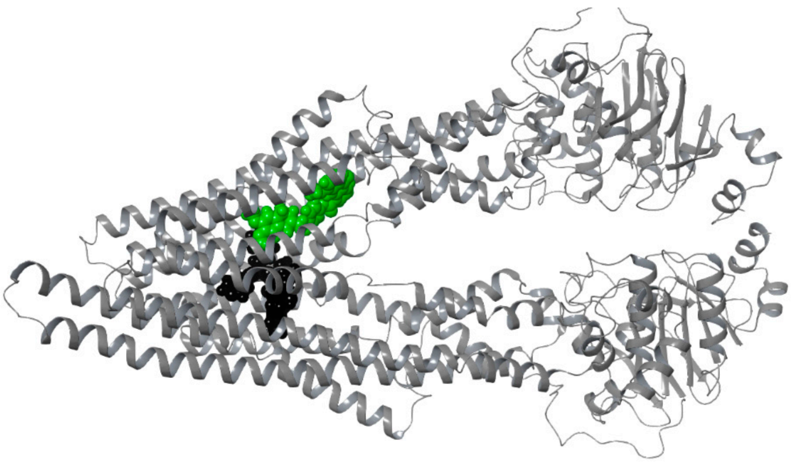

(b)

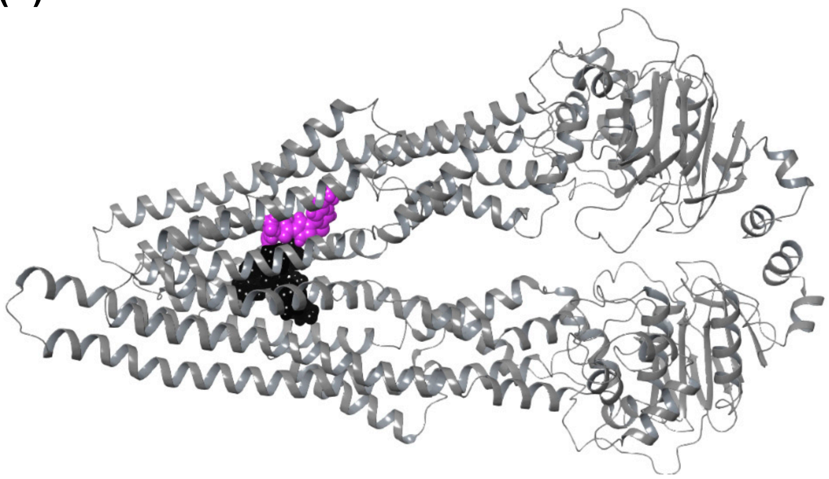

(c)

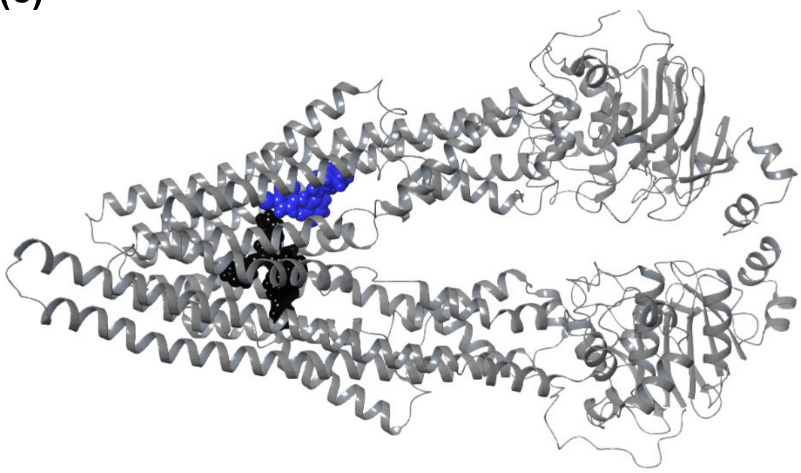

Supplement: Supplementary file 1 [file ijms-26-11448-s001.zip › Figures S1-S5.pdf]
