# Supplementary material for: Targeting the MEK/ERK Pathway to Suppress P-Glycoprotein and Reverse Carfilzomib Resistance in Multiple Myeloma
Source: Int J Mol Sci. 2025 Nov 26;26(23):11448. doi: 10.3390/ijms262311448 (PMC12692345; doi:10.3390/ijms262311448)
Supplement: Supplementary file 1 [file ijms-26-11448-s001.zip › Supplementary Tables S1-S4.pdf]

Supplementary Table S1. Values of half maximal inhibitory concentrations (IC_{50}, μM) and low-toxicity concentrations of ulixertinib and cobimetinib for AMO-1 and AMO-1/CFZ cells.

	AMO-1			AMO-1/CFZ		
	**IC_{50}, μM**	**Low-toxicity concentration**		**IC_{50}, μM**	**Low-toxicity concentration**	
		μM	cells viability (%)		μM	cells viability (%)
Cobimetinib	12.4 ± 4.0	1	92.9 ± 4.6	14.1 ± 2.0	1	99.1 ± 4.7
Ulixertinib	31.5 ± 0.5	10	96.7 ± 7.1	35.5 ± 0.7	10	86.9 ± 10.3

Supplementary Table S2: Evaluation of the efficiency of Rhodamine 123 (Rh123) release from AMO-1/CFZ cells in the presence of elacridar, cobimetinib, ulixertinib, or carfilzomib (mean and SD, 3 biological replicates).

	**Fluorescent cells (%, mean)**	**SD**
Enter of Rh123	97.8	0.9
Efflux of Rh123 in pure medium	13.9	5.7
Efflux of Rh123 in the presence Elacridar 1 μM	97.8	2.8
Efflux of Rh123 in the presence Elacridar 10 μM	99.1	1.2
Efflux of Rh123 in the presence Cobimetinib 1 μM	10.5	3.2
Efflux of Rh123 in the presence Cobimetinib 20 μM	87.1	11.7
Efflux of Rh123 in the presence Ulixertinib 1 μM	9.5	3.0
Efflux of Rh123 in the presence Ulixertinib 10 μM	87.6	6.8
Efflux of Rh123 in the presence Ulixertinib 20 μM	92.5	6.5
Efflux of Rh123 in the presence Carfilzomib 0.5 μM	8.1	2.6
Efflux of Rh123 in the presence Carfilzomib 20 μM	28.9	2.9

Supplementary Table S3. *In silico* molecular docking parameters for P-glycoprotein Interaction with well-known substrates and inhibitors of P-gp and two proteasome inhibitors.

**Substance name**	**Substance class in relation to P-glycoprotein**	**Docking score**	**Position in the molecule**	**Interaction**
Paclitaxel (PDB: TA1)	Substrate	-8.226	Center	H-bond (4) GLU875, GLN990, TYR310
Doxorubicin (PubChem: 31703)	Substrate	-7.483	Center	Pi-pi stacking (2), H-bond TRP232, GLU875
Rhodamine 123 (PubChem: 65218)	Substrate	-5.127	Center	-
Verapamil (PubChem: 2520)	Inhibitor	-6.139	Radially	Pi-pi stacking, H-bond (2), Pi-cation PHE770, GLN838, LYS826
Tariquidar (PubChem: 148201)	Inhibitor	-10.398	Radially	Pi-pi stacking (2) PHE303, PHE770
Ixazomib (PDB: 6V8)	-	-7.459	Radially	H-bond ASN296
Bortezomib (PDB: BO2)	-	-7.437	Radially	Pi-pi stacking, H-bond (4) GLN725, GLN990, TRP232

Supplementary Table S4. Real-time PCR primer sequences.

Genes	Forward primer	Reverse primer
RPLPO	CCTTCTCCTTTGGGCTGGTCATCCA	CAGACACTGGCAACATTGCGGACAC
ABCB1	GGGATGGTCAGTGTTGATGGA	GCTATCGTGGTGGCAAACAATA
EPHB2	CTACTGTAACGGGGACGGC	CCAGATGGACAACCTCGGC
IL6R	GGGTTGTGGAATCTTGCAGC	TGTCTTTGACCGTTCAGCCC
CROT	TGGCTAAATCAACTGAAGAACGAAC	CAAGTGAAGGAACAGGCAGTG
